# Utilization of central nervous system resources for preparation and performance of complex walking tasks in older adults

**DOI:** 10.3389/fnagi.2014.00217

**Published:** 2014-08-25

**Authors:** David J. Clark, Dorian K. Rose, Sarah A. Ring, Eric C. Porges

**Affiliations:** ^1^Brain Rehabilitation Research Center, Malcom Randall VA Medical Center, North Florida/South Georgia Veterans Health SystemGainesville, FL, USA; ^2^Department of Aging and Geriatric Research, University of FloridaGainesville, FL, USA; ^3^Department of Physical Therapy, University of FloridaGainesville, FL, USA

**Keywords:** walking, aging, motor control, near infrared spectroscopy, skin conductance

## Abstract

**Introduction:** Walking in the home and community often involves performance of complex walking tasks. Understanding the control of such tasks is crucial to preserving independence and quality of life in older adults. However, very little research has been conducted in this area. Here, we assess the extent to which two measures of central nervous system (CNS) activity are responsive to the challenges posed by preparation and performance of complex walking tasks. Prefrontal cortical activity was measured by functional near-infrared spectroscopy (fNIRS) and sympathetic nervous system arousal was measured by skin conductance level (SCL).

**Materials and methods:** Sixteen older men and women (age: 77.2 ± 5.6 years) with mild mobility deficits participated in this study. Participants walked at their preferred speed without distractions along an unobstructed, well-lit course (*control* task) and also walked on the same course under five separate challenging conditions: performing a cognitive verbal fluency task (*verbal* task), dim lighting (*dim* task), carrying a tray (*carry* task), negotiating obstacles (*obstacles* task) and wearing a weighted vest (*vest* task). Mean prefrontal activation and SCL were calculated during the preparation and performance phases of each task. Gait spatiotemporal measurements were acquired by an instrumented gait mat.

**Results:** Prefrontal cortical activity and SCL were elevated during the preparation phase of complex walking tasks relative to the control task. During the performance phase, prefrontal activity remained elevated to a similar level as during task preparation. In contrast, SCL continued to increase beyond the level observed during task preparation. A larger increase in prefrontal activity was found to be linked to preserved quality of gait during complex walking tasks.

**Discussion:** These findings indicate that availability and utilization of CNS resources are important for optimizing performance of complex walking tasks in older adults.

## INTRODUCTION

Walking in the home and community environment often involves performance of complex tasks (Patla and Shumway-Cook, 1999; Frank and Patla, 2003; Lord et al., 2010). Such tasks can encompass a variety of different conditions such as walking over obstacles, in low-lighting and while multi-tasking. Compared to typical steady-state walking, these tasks require heightened demand on motor and/or cognitive resources. A study of over 1200 older adults by Shumway-Cook et al. (2007) demonstrated that age-related performance decrements are more severe for complex walking tasks than for typical steady-state walking. Furthermore, age-related mobility disability has been shown to be characterized in part by avoidance of environments that require performance of complex walking tasks (Shumway-Cook et al., 2002, 2003). There is a crucial need for enhanced understanding of the control of complex walking tasks in older adults. This knowledge will contribute to the development of novel mobility assessments and to the design of new therapeutic interventions that seek to preserve independent ambulation in the home and community.

Very little research has been conducted to understand the central nervous system (CNS) determinants of complex walking task performance, especially in older adults. The present study sought to advance this area of research by examining prefrontal cortical activity and sympathetic nervous system (SNS) arousal during the preparation and performance phases of various complex walking tasks. Prefrontal cortical activity was quantified by functional near-infrared spectroscopy (fNIRS). fNIRS is a relatively new technology that non-invasively assesses tissue metabolic activity using near-infrared light, which is able to pass through bone and other biological tissues (Bunce et al., 2006). Metabolic activity is estimated based on the characteristic properties of infrared light absorption by oxygenated hemoglobin and deoxygenated hemoglobin (markers of oxygen delivery and utilization) (Bunce et al., 2006). Prefrontal activation has been shown to be broadly linked to the amount of attention/intention directed toward cognitive and motor tasks, including walking (Okamoto et al., 2004; Herrmann et al., 2006; Suzuki et al., 2008; Atsumori et al., 2010; Kaneko et al., 2011; Koenraadt et al., 2014). In some studies, higher levels of prefrontal activity have been linked to better performance under novel task conditions (Ohsugi et al., 2013; Ishikuro et al., 2014).

SNS arousal was quantified by skin conductance level (SCL), which measures the electrical conductance of the skin. SCL is affected by sweat gland activity, which is under the control of the SNS (Sato et al., 1989). One function of the SNS is to mobilize physiological resources (e.g., increase blood flow and glucose release) under physically or psychologically challenging conditions (Bear et al., 2007). It has been proposed to up-regulate the gain of the entire CNS to increase responsiveness (Sibley et al., 2010). Although increased arousal can be beneficial to task performance under certain conditions, excessive arousal is known to compromise performance (Yerkes and Dodson, 1908). The extent to which this may affect mobility performance in older adults remains unclear. It is possible that fear of falling due to the challenge of walking with an aged CNS and musculoskeletal system could lead to excessively heightened arousal. For instance, heightened arousal/anxiety due to fear of falling has previously been linked to abnormal responses for posture (Adkin et al., 2002; Carpenter et al., 2004) and gait (Brown et al., 2002; Hadjistavropoulos et al., 2012).

Our primary objective was to assess the extent to which prefrontal activity and SCL were responsive to the increased utilization of CNS resources that is expected to occur when preparing for and executing complex walking tasks. Our secondary objective was to assess the potential link between CNS responses and gait quality during performance of the complex walking tasks. We hypothesized that prefrontal activity (interpreted as utilization and availability of cognitive resources) would be increased during preparation and performance of complex walking tasks, and that higher responses would be linked to better gait performance. We also hypothesized that SCL (interpreted as cognitive load and arousal) would be increased during preparation and performance of complex walking tasks, but that higher responses would be linked to poorer gait performance.

## MATERIALS AND METHODS

### PARTICIPANTS

Men and women between the ages of 65–85 years with mild mobility deficits were recruited for this study. Mild mobility deficits were defined as 400 m walking speed less than 1.1 m/s and agreement with the statement “You find it physically tiring to walk a quarter mile, or climb two flights of stairs, or perform household chores.” Preliminary screening of inclusion/exclusion criteria was conducted by telephone. Exclusion criteria included use of an assistive device for walking (cane, crutch, walker, brace, etc.); lower extremity pain while walking; involuntary weight gain or loss exceeding 10 pounds within the past six months; myocardial infarction or symptomatic cardiovascular disease in the past year; bone fracture in the past year; injury or illness to the CNS; uncontrolled hypertension exceeding 160 systolic and/or 95 diastolic; or terminal illness. Volunteers who met these criteria were invited to our research center for further screening. Additional inclusion criteria applied at the onsite visit were Berg Balance Scale (BBS, Berg et al., 1989) score ≥ 41, Mini-Mental State Exam (MMSE, Folstein et al., 1975) score ≥ 21, and body mass index (BMI) within the range of 19–35. All study procedures were approved by the University of Florida Institutional Review Board. All individuals provided their written informed consent at time of enrollment.

### PROTOCOL AND EQUIPMENT

The assessments described here are a subset from a larger experimental protocol (PI: David J. Clark) that tested motor control of walking in older adults. The study was conducted over three separate visits to our research center. The first day consisted of functional assessments and questionnaires. This included the screening items mentioned in the prior section (BBS and MMSE) as well as the Activities-Specific Balance Confidence Scale (ABC Scale). The second and third day involved mechanistic assessments of walking including fNIRS of the prefrontal cortex, SCL and spatiotemporal gait measurement. The overground walking course consisted of five consecutive laps around an 18 m course in our laboratory, for a total walking distance of 90 m. The floor surface was smooth tile with the exception of the 5.2 m instrumented walkway, which has the texture of firm foam (GAITRite, CIR Systems, Sparta, NJ, USA). Participants walked at their preferred speed without distractions along an unobstructed, well-lit pathway (*control* task) and also walked on the same course while separately performing the following five additional challenging tasks on separate trials: cognitive verbal fluency task (“*verbal*”), dim lighting (“*dim*”), carrying a tray (“*carry*”), negotiating obstacles (“*obstacles*”) and wearing a weighted vest (“*vest*”). The *control*, *verbal*, *dim,* and *carry* tasks were conducted on the second visit to our center, and the *control*, *obstacle,* and *vest* tasks were conducted on the third visit. For the *verbal* task, participants were asked to say as many words as possible that began with a randomly selected letter. In order to maintain a comparable level of cognitive effort over the duration of the trial, a new letter was provided to the participant for each lap. For the *dim* task the lights in the laboratory were turned off and the windows were blocked, but some light entered through the door of an adjoining room. The participant’s eyes were allowed to adjust for approximately 2 min prior to starting the task. For the *carry* task, three rolls of athletic tape were stacked on the tray, and the participant was instructed to keep the tray stable to avoid having the stack fall over. For the *obstacle* task, the participant was instructed to step over six small obstacles (shoes) evenly spaced along the walking path. For the *vest* task, the participant wore an adjustable weighted vest with a load equal to 10% of body weight. The load was evenly distributed between the front and rear of the vest, and velcro straps were used to ensure that the vest remained snug against the body. Prior to performing each task, the participant stood quietly at the start of the course for about one minute to allow measurement of CNS activity during task preparation. The participants were given no specific instructions regarding how to perform or prioritize the objectives within each task, because our goal was to evaluate their natural/preferred walking behavior.

Metabolic activity of the left and right anterior prefrontal cortices (Brodmann Area 10) was evaluated with a commercially available fNIRS monitor (Niro 200NX, Hamamatsu Photonics, Japan). In accordance with prior studies, each set of probes (i.e., for left and right prefrontal cortices) were placed high on the forehead to avoid the temporalis muscle (but not over hair) and sufficiently lateral from the midline to avoid the superior sagittal sinus (Al-Rawi and Kirkpatrick, 2006; Tisdall et al., 2009). Optode spacing was 3 cm. fNIRS uses light of varying wavelengths to non-invasively record changes in blood flow and hemoglobin due to neuronal activation of cerebral cortex. SCL was measured using a commercially available data acquisition unit (Flexcomp Infiniti, Thought Technology Ltd., QC, Canada) and sensors (SA9309M, Thought Technology Ltd., QC, Canada). Sensors were secured to the proximal phalanges of the index and ring fingers using a velcro strap. SCL signals were acquired separately from both the left and right hands. fNIRS and SCL data were sampled at 2 and 32 Hz, respectively, and saved to a memory card in each data acquisition unit. Spatiotemporal gait data were acquired from the instrumented walkway.

### DATA ANALYSIS

Raw data were converted into text files via each manufacturer’s software, then analyzed with custom programs in Matlab version R2011b (The Mathworks, Natick, MA, USA). Prefrontal cortical activity was quantified as the tissue oxygenation index (TOI), which is the ratio (expressed as a percent) of oxygenated hemoglobin to total hemoglobin (sum of oxygenated and deoxygenated). TOI provides a real-time measure of the balance between cerebral oxygen delivery and utilization (Tisdall et al., 2009). For both TOI and SCL, the average magnitude was calculated for a 10-s epoch in which the participants were standing still immediately preceding task performance (preparation phase) and also for the full period of steady-state walking (performance phase). The transition periods for gait initiation and gait termination were excluded from the analysis. Gait variables extracted from the instrumented walkway included measures typically used to infer the use of a cautious gait pattern: decreased speed, increased stance width, increased double limb support time and increased variability (measured by standard deviation of step length and double support time).

### STATISTICS

Statistical analysis was conducted with JMP Pro software (version 11.0.0). Two-way repeated-measures ANOVA models were used to assess task- and side-dependent differences in prefrontal TOI (task × hemisphere) and SCL (task × hand). Separate models were used for tasks that were assessed on the first day of evaluation (*control*, *verbal, dim* and *carry*) and the second day of evaluation (*control, obstacles*, and *vest*). Mauchly’s sphericity test was used to test the assumption of sphericity in the ANOVA models. If sphericity was not achieved, the model was adjusted using the Huynh–Feldt correction (if 𝜖 > 0.75) or the Greenhouse–Geisser correction (if 𝜖 < 0.75). *Post hoc* testing of significant main effects was conducted using one-sided repeated-measures *t*-tests to test the hypothesis that complex walking tasks increased TOI and SCL relative to the preparation phase of each task and/or relative to performance of the *control* walking task. The false discovery rate procedure (Curran-Everett, 2000; Curran-Everett and Benos, 2004) was used to correct for multiple comparisons during *post hoc* testing. Secondary analyses were conducted to determine whether there was evidence to support a link between CNS responses and gait quality. For TOI, separate two-tailed *t*-tests were used to test the hypothesis that gait quality would be better in a high-response subgroup versus a low-response subgroup. For SCL, separate two-tailed *t*-tests were used to test the hypothesis that gait quality would be better in a low-response subgroup versus a high-response subgroup.

## RESULTS

### PARTICIPANTS

Sixteen older adults (8 male/8 female) participated in this study. The mean age of participants was 77.2 ± 5.6 years, with a range of 66–85 years. Additional information from clinical assessments is presented in **Table 1**.

**Table 1:** Participant characteristics.

	Mean	Range
Body Mass Index (kg/m^2^)	29.3 ± 2.7	24.3–33.8
Mini Mental State Exam	27.4 ± 1.7	24–30
Berg Balance Test	50.5 ± 3.9	42–56
400 m Walk speed (m/s)	0.92 ± 0.11	0.66–1.08
ABC Scale (% confidence)	83.0 ± 15.0	58–100

### fNIRS

**FIGURE 1:**
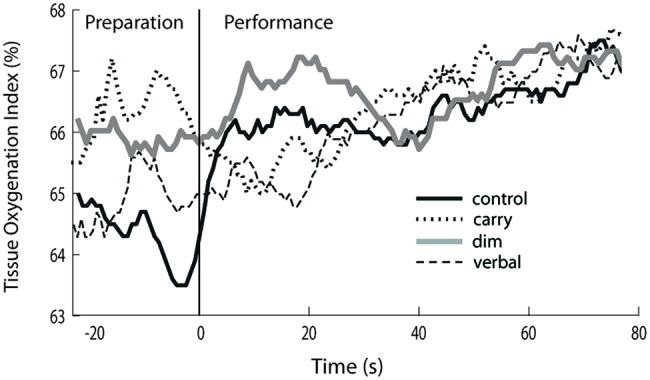
**Exemplar data demonstrating task-dependent differences in tissue oxygenation index (TOI).** Prefrontal cortical activation, as indicated by TOI, for one study participant. Data are shown for walking along an unobstructed, well-lit pathway (*control* task; black solid line), walking while carrying a tray (*carry* task; dotted line), walking in dim lighting (*dim* task; gray solid line) and walking while performing a verbal fluency task (*verbal* task; dashed line). The participant is standing still for a short period of time at the start of the trial (preparation phase) and then walks at preferred speed (performance phase).

**FIGURE 2:**
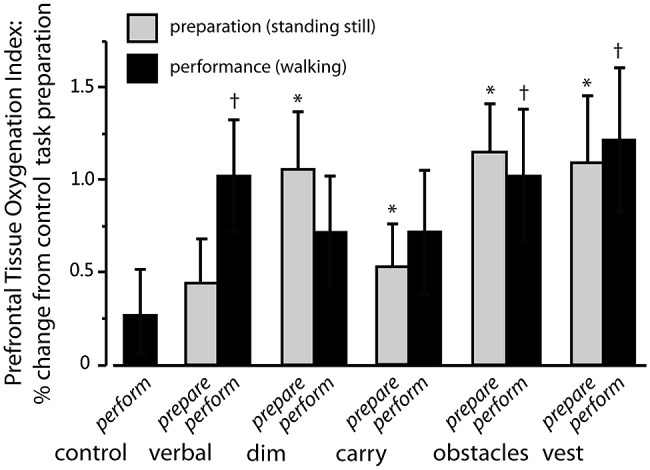
**Increase in prefrontal activation for complex tasks relative to preparation for control task.** Gray bars indicate the average percentage increase in prefrontal activation during the preparation phase of each complex walking task relative to preparation for the *control* task. Significant changes are shown by an asterisk. Black bars indicate the percentage increase in prefrontal activation during the performance phase of each complex walking task relative to preparation for the *control* task. Significant changes are shown by a dagger. Error bars indicate group standard error.

Exemplar fNIRS data for one participant are shown in **Figure 1** and group average data are shown in **Figure 2**. For **Figure 2**, the TOI value shown for the performance phase of the control task is the average of the values from visit 1 and visit 2. These values did not differ significantly across visits (0.29 ± 1.13 versus 0.48 ± 1.24, respectively, *p* = 0.38).

#### Preparation for complex walking tasks

For TOI during the preparation phase preceding each walking task, there was a significant main effect of task (*p* < 0.05). The size of that effect across tasks is shown by the gray bars in **Figure 2**, which show the percent increase in TOI for the preparation phase of each complex walking task relative to the TOI for the preparation phase of the *control* task. *Post hoc* analysis revealed that, relative to *control*_prepare_, TOI increased significantly for *dim*_prepare_ (*p* = 0.003), *carry*_prepare_ (*p* = 0.04), *vest*_prepare_ (*p* = 0.006) and *obstacles*_prepare_ (*p* = 0.001). There was also a trend toward significance for *verbal*_prepare_ (*p* = 0.09). The effects of hemisphere and task × hemisphere interaction were not significant.

#### Performance of complex walking tasks

For TOI during the performance phase of each task, a significant main effect of task was also found (*p* < 0.05). The size of that effect across tasks is indicated by the black bars of **Figure 2**, which show the percent increase in TOI for the performance phase of each complex walking task relative to the TOI for the preparation phase of the *control* task. *Post hoc* analysis revealed that, relative to *control*_prepare_, TOI increased for *verbal*_perform_ (*p* = 0.006), *vest*_perform_ (*p* = 0.005), and *obstacles*_perform_ (*p* = 0.002). There was a trend toward increased TOI during *dim*_perform_ (*p* = 0.06) and carry_perform_ (*p* = 0.11). The effects of hemisphere and task × hemisphere interaction were not significant.

#### Within-task comparison of preparation versus performance

Also examined was whether TOI increased from the preparation phase to the performance phase within each task (i.e., comparing gray bars to black bars for each task in **Figure 2**). TOI for preparation versus performance did not increase significantly between *dim*_prepare_ and *dim*_perform_ (*p* = 0.95), between *carry*_prepare_ and *carry*_perform_ (*p* = 0.16), between *obstacles*_prepare_ and *obstacles*_perform_ (*p* = 0.84), or between *vest*_prepare_ and *vest*_perform_ (*p* = 0.30). However, there was a trend for increased TOI between *verbal*_prepare_ and *verbal*_perform_ (*p* = 0.05).

### SKIN CONDUCTANCE LEVEL

**FIGURE 3:**
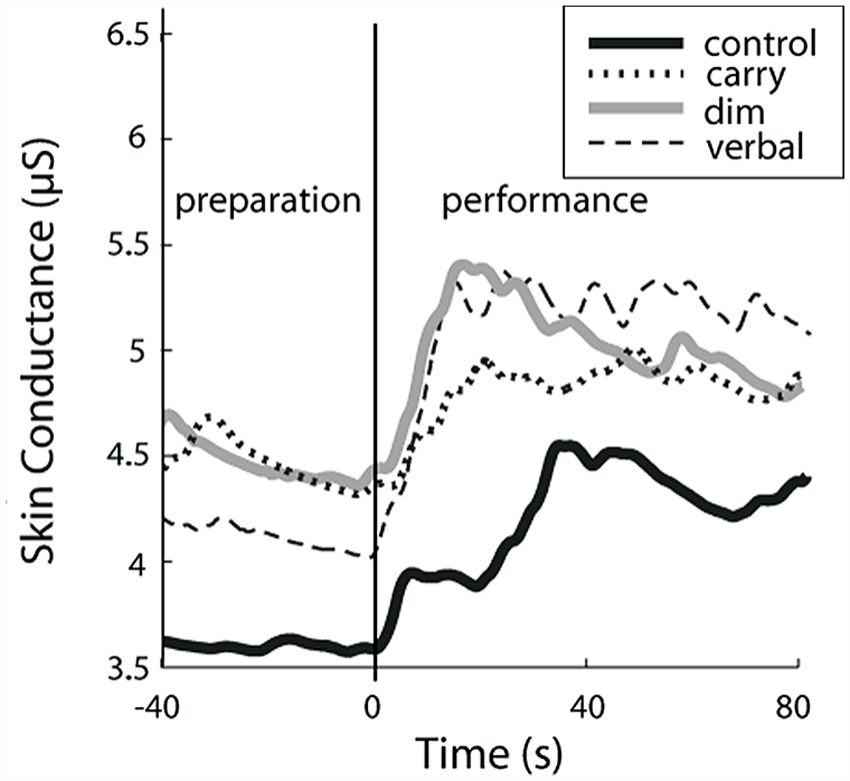
**Exemplar data demonstrating task-dependent differences in skin conductance level (SCL).** Sympathetic nervous system (SNS) arousal, as indicated by SCL, for one study participant. Data are shown for walking along an unobstructed, well-lit pathway (*control* task; black solid line), walking while carrying a tray (*carry* task; dotted line), walking in dim lighting (*dim* task; gray solid line) and walking while performing a verbal fluency task (*verbal* task; dashed line). The participant is standing still for a short period of time at the start of the trial (preparation phase) and then walks at preferred speed (performance phase).

**FIGURE 4:**
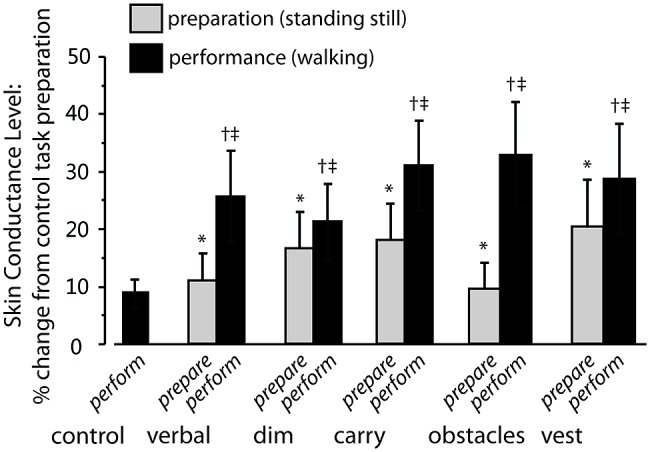
**Increase in skin conductance level (SCL) for complex tasks relative to preparation for control task.** Gray bars indicate the average percentage increase in SCL during the preparation phase of each complex walking task relative to preparation for the *control* task. Significant changes are shown by an asterisk. Black bars indicate the percentage increase in SCL during the performance phase of each complex walking task relative to preparation for the *control* task. Significant changes are shown by a dagger. Significant within-task differences (preparation versus performance) are shows by a double dagger. Error bars indicate group standard error.

Exemplar SCL data for one participant are shown in **Figure 3** and group average data are shown in **Figure 4**. For **Figure 4**, the SCL value shown for the performance phase of the control task is the average of the values from visit 1 and visit 2. These values did not differ significantly across visits (8.02 ± 11.48 versus 9.85 ± 10.74, respectively, *p* = 0.65).

#### Preparation for complex walking tasks

For SCL during the preparation phase of each task, there was a significant main effect of task (*p* < 0.05). The size of that effect across tasks is shown by the gray bars in **Figure 4**, which show the change in SCL for the preparation phase of each complex walking task relative to the TOI for the preparation phase of the *control* task. *Post hoc* analysis revealed that, relative to *control*_prepare_, SCL increased significantly for *verbal*_prepare_ (*p* = 0.03), *dim*_prepare_ (*p* = 0.04), *carry*_prepare_ (*p* = 0.02), *obstacles*_prepare_ (*p* = 0.02), and *vest*_prepare_ (*p* = 0.03). The effects of side (right and left hands) and task × side interactions were not significant.

#### Performance of complex walking tasks

For SCL during the performance phase of each task, a significant main effect of task was also found (*p* < 0.05). The size of that effect across tasks is indicated by the black bars of **Figure 4**, which show the change in SCL for the performance phase of each complex walking task relative to the SCL for the preparation phase of the *control* task. *Post hoc* analysis revealed that, relative to *control*_prepare_, SCL increased significantly for *verbal*_perform_ (*p* = 0.02), *dim*_perform_ (*p* = 0.04), *carry*_perform_ (*p* = 0.01), *obstacles*_perform_ (*p* = 0.02), and *vest*_perform_ (*p* = 0.03). The effects of side (right and left hands) and task × side interaction were not significant.

#### Within-task comparison of preparation versus performance

Also examined was whether SCL increased from the preparation phase to the performance phase within each task (i.e., comparing gray bars to black bars for each task in **Figure 4**). Preparation and performance for TOI increased significantly between *dim*_prepare_ to *dim*_perform_ (*p* = 0.001), between *verbal*_prepare_ and *verbal*_perform_ (*p* < 0.001), between *carry*_prepare_ and *carry*_perform_ (*p* < 0.001), between *vest*_prepare_ and *vest*_perform_ (*p* < 0.001), and between *obstacles*_prepare_ and *obstacles*_perform_ (*p* < 0.001).

### GAIT SPATIOTEMPORAL MEASUREMENTS

Gait spatiotemporal measurements were adversely affected by the complex walking tasks. Relative to *control*, walking speed was reduced for *carry* (-0.05 m/s, *p* = 0.02), *verbal* (-0.15 m/s, *p* < 0.001), *obstacles* (-0.15 m/s, *p* < 0.001) and tended to be reduced for *dim* (-0.02 m/s, *p* = 0.09). Base of support was increased for *carry* (0.89 cm, *p* = 0.03), *verbal* (0.82 cm, *p* = 0.008), and *dim* (0.94 cm, *p* = 0.005). Double support % was increased for *carry* (2.2%, *p* = 0.002), *verbal* (2.5%, *p* < 0.001), and *vest* (0.7%, *p* = 0.02). Variability of step length and double support time increased for *obstacles* (as expected due to the irregular terrain).

Secondary analyses were conducted to determine if the size of the response for prefrontal activation and/or SCL during the performance phase of complex walking tasks was related to the severity of the gait disturbance. For each CNS measure, the study sample was split in half to form two subgroups: low-response subgroup and high-response subgroup. For prefrontal activation, the “response” was calculated as the change in TOI that occurred between the preparation of the *control* task and the performance phase of each complex walking task. For each individual, the average response across all complex walking tasks was calculated. The low-response subgroup for TOI had an average change of -0.2 ± 1.1 percentage points (i.e., roughly unchanged prefrontal activity) and the high-response subgroup had a mean change in TOI of 2.1 ± 0.6 percentage points (i.e., increased prefrontal activity for complex tasks). For each TOI subgroup, average gait measurements during the *control* task are reported in **Table 2A**. The subgroups did not differ significantly for any *control* gait variable, with the exception of a trend for a wider base of support in the high-response subgroup. The subgroups also did not differ significantly for age, body mass index, or MMSE score (all p-values greater than 0.53). Significant differences between subgroups (or a trend) indicate that the high response subgroup had a smaller reduction of walking speed for *obstacles* (*p* = 0.003) and *vest* (*p* = 0.001); lower proportion of gait cycle spent in double limb support for *obstacles* (*p* = 0.10) and *vest* (*p* = 0.04); and less increase for step length variability for *carry* (*p* = 0.02), and *dim* (*p* = 0.04). Although the effect of complex walking tasks was not statistically different between groups for most gait variables, it may be valuable to note that the direction of differences favored the high response subgroup as having less gait disturbance for 76% of the variables (19 out of 25; as indicated in **Table 3**). The mean effect size for this subset of gait variables was 0.76 ± 0.54.

**Table 2:** Control task gait values for TOI and SCL response subgroups.

	High	Low	*p*
**(A) TOI response subgroups**
Walking speed (m/s)	97.5 ± 9.5	102.5 ± 11.3	0.44
Base of support (cm)	12.5 ± 3.1	8.1 ± 2.7	0.04
Double support (% gait cycle)	34.1 ± 1.5	34.4 ± 2.8	0.85
Step length variability*	2.81 ± 2.10	1.66 ± 0.43	0.26
Double support time variability**	0.022 ± .009	0.021 ± .006	0.75
**(B) SCL response subgroups**
Walking speed (m/s)	102.7 ± 14.0	95.9 ± 15.1	0.42
Base of support (cm)	9.9 ± 4.0	12.8 ± 3.0	0.18
Double support (% gait cycle)	34.5 ± 1.8	35.3 ± 3.5	0.59
Step length variability*	2.47 ± 2.04	2.4 ± 1.19	0.94
Double support time variability**	0.02 ± .009	0.026 ± .010	0.26

*Units are in cm SD. **Units are % gait cycle SD.

**Table 3:** Percentage change in gait variables for TOI response subgroups.

	High response	Low response	Effect size	
**Walking speed**
Carry	0.0 ± 7.3	-3.4 ± 5.5	0.51	0.40	
Dim	0.6 ± 7.3	4.4 ± 5.3	0.61	0.35	
Verbal	-13.6 ± 8.3	-12.5 ± 9.1	0.13	0.85	
*Obstacles*	-*5.5 ± 6.2*	-*21.3 ± 5.8*	*1.72*	*0.003*	
*Vest*	*7.0 ± 3.4*	-*5.3 ± 4.3*	*1.83*	*0.001*	
**Base of support**
Carry	6.9 ± 24.9	12.1 ± 15.8	0.28	0.69	
Dim	11.1 ± 15.4	16.3 ± 18.3	0.34	0.63	
Verbal	11.9 ± 9.8	0.7 ± 15.1	0.92	0.20	
Obstacles	8.3 ± 24.0	6.8 ± 19.2	0.07	0.92	
Vest	4.3 ± 20.0	-5.1 ± 18.3	0.54	0.46	
**Double support time**
Carry	1.0 ± 1.4	1.6 ± 2.7	0.27	0.67	
Dim	0.9 ± 0.8	-0.1 ± 0.8	1.07	0.06	
Verbal	2.1 ± 1.3	2.6 ± 2.5	0.26	0.69	
Obstacles	-6.8 ± 1.9	-4.3 ± 2.2	1.16	0.10	
V*est*	-*0.2 ± 0.5*	*1.4 ± 1.2*	*1.36*	*0.04*	
**Step length variability**
*Carry*	-*4.3 ± 31.6*	*55.0 ± 37.7*	*1.39*	*0.02*	
*Dim*	-*13.8 ± 35.8*	*16.2 ± 33.1*	*1.18*	*0.04*	
Verbal	1.49 ± 50.6	63.6 ± 39.1	0.81	0.18	
Obstacles	270.7 ± 184.1	376.9 ± 220.5	0.54	0.43	
Vest	1.3 ± 42.6	20.0 ± 30.5	0.56	0.45	
**Double support time Variability**
Carry	-0.2 ± 30.1	44.8 ± 55.4	1.04	0.16	
Dim	-0.5 ± 41.7	7.3 ± 37.9	0.22	0.75	
Verbal	25.6 ± 47.2	65.9 ± 125.7	0.49	0.53	
Obstacles	77.8 ± 77.3	91.6 ± 92.2	0.18	0.80	
Vest	2.5 ± 33.8	13.7 ± 43.9	0.32	0.66	

*For each complex walking task, the percentage change for each gait variable is calculated relative to the control task. Results are shown for the subgroups determined by higher and lower response of prefrontal cortical activation. Italicized items indicate statistical significance as determined by two-tailed *t*-tests (*p* < 0.05). All values are mean ± SD.*

The same type of exploratory subgroup analysis as just described was conducted for SCL. The low-response subgroup had an average change in SCL of -2.9 ± 4.2% (i.e., roughly unchanged SNS arousal) and the high-response subgroup had a mean change in SCL of 47.7 ± 29.7% (i.e., increased SNS arousal for complex tasks). For each SCL subgroup, average gait measurements during the *control* task are reported in **Table 2B**. The subgroups did not differ significantly for any *control* gait variable. The low and high SCL response subgroup differed significantly for age (73.6 versus 81.3 years, *p* = 0.005), but not for body mass index (*p* = 0.24) or MMSE score (*p* = 0.74). Accordingly, the gait variables were corrected for age prior to evaluating the effect of complex walking tasks. None of the age-corrected gait variables were found to differ significantly between the low and high SCL response subgroups (all *p*-values ≥ 0.23). Also examined was the potential association between the mean response size for prefrontal activity and the mean response size for SCL (means calculated across all tasks), in order to investigate the presence of a link between these physiological mechanisms. No association was found (*p* = 0.90).

## DISCUSSION

Performance of complex walking tasks is adversely affected by aging (Shumway-Cook et al., 2007), which is concerning given the high relevance of such tasks to everyday ambulation in the home and community. Our choice of tasks was motivated by the “*person-environment interaction model”* of mobility proposed by Patla and Shumway-Cook (1999) and Shumway-Cook et al. (2002). This model describes multiple dimensions that must be mastered to attain successful community ambulatory function. Four dimensions are represented by the tasks in this study: terrain (*obstacles* task), physical load (*vest* task), ambient conditions (*dim* task), and attentional demands (*verbal* and *carry* tasks). The results of this study reveal that prefrontal cortical activity and SNS arousal are elevated during both the preparation and performance phases of complex walking tasks. Furthermore, the changes in prefrontal activity during performance of complex walking tasks were found to be linked to the quality of gait. These findings suggest that utilization of CNS resources is increased to optimize performance of complex walking tasks, and that a shortage of available resources contributes to performance decrements in some older adults.

Prefrontal cortical activation is important to complex walking tasks because of its role in preparing for and executing purposeful actions (Fuster, 2000; Jenkins et al., 2000; Tanji and Hoshi, 2001), including walking (Suzuki et al., 2008; Atsumori et al., 2010; Koenraadt et al., 2014). Our results show that prefrontal TOI increased bilaterally during preparation for all complex walking tasks with the exception of the *verbal* task. During the performance phase of the complex walking tasks, prefrontal TOI remained elevated but generally did not exceed the magnitude observed during the preparation phase. Again, the only notable exception was for *verbal*, where TOI was more strongly elevated during performance than during preparation. This is in agreement with the strongly increased demand for prefrontal resources that is associated with cognitive processing (Holtzer et al., 2011; Ohsugi et al., 2013). Our secondary analysis of subgroups with low versus high prefrontal response generally supported our hypothesis that a high response is linked to better gait performance. Most gait variables (19 out of the 25 when accounting for all tasks) seemed to favor the high-response subgroup, although only a small number were found to be statistically significantly because the analysis was underpowered. For these 19 variables, the average effect size was 0.76, which is considered to be a large effect (Cohen, 1988). Although these results are preliminary, the possible advantage for the high-response subgroup over the low-response subgroup provides encouraging rationale for pursuing this mechanistic finding in future research. We propose that a high response for prefrontal TOI indicates an available reserve supply of prefrontal resources that can contribute to control of complex walking tasks. This reserve supply helps to prevent against excessive competition for central neural resources, thereby preserving movement quality. This assertion is generally consistent with the “supply and demand framework” described by Seidler et al. (2010). These findings build upon prior work that has demonstrated an association between structural integrity of the cortex and gait performance under non-challenging walking conditions (Rosso et al., 2013; Holtzer et al., 2014). For instance, less cerebral gray matter volume of the prefrontal, medial temporal, frontoparietal and sensorimotor areas has been linked to walking decrements such as slow speed, shorter steps, and longer double support time (Rosano et al., 2007, 2008, 2012). The presence of cerebral white matter hyperintensities have also been shown to be linked to decrements in walking ability (Rosano et al., 2010; Wakefield et al., 2010; Viana-Baptista et al., 2011; Moscufo et al., 2012; Willey et al., 2013).

We quantified SNS arousal using the well-established measure of SCL. Our results show that SCL increases substantially when preparing for the complex walking tasks and increases even further when performing the complex walking tasks. However, our hypothesis of a link between SCL and gait performance during complex walking tasks was not supported. Future research is warranted to determine whether a link between SCL response and gait performance may be revealed by comparing cohorts with different characteristics (such as low versus high functioning elders). No association was found between the magnitude of SCL and prefrontal responses. The difference in SCL and prefrontal responses was also evident in the pattern of change that was observed across testing conditions. The elevation in SCL occurred in a stepwise manner, such that (1) SCL for complex task preparation increased over *control* task preparation, and (2) SCL for complex task performance increased over complex task preparation. In agreement with SCL, prefrontal TOI for complex task preparation increased over *control* task preparation. However, in contrast to SCL, no further increase was seen between complex task preparation and complex task performance. Cumulatively, our findings suggest that SCL and prefrontal TOI represent different functions of the CNS for control of walking.

An important finding from our data is that CNS assessments provide the advantage of being more broadly responsive to neurophysiological demand than any single spatiotemporal measure of gait performance. The spatiotemporal gait parameters that were affected by complex walking tasks differed depending on the task. Likewise, the gait parameters that were linked to prefrontal TOI response also differed depending on the task. This finding is not particularly surprising, because the gait modification that is adopted by an individual can be expected to depend on task objectives and also on what facet of performance is prioritized (Al-Yahya et al., 2009; Hobert et al., 2011; Oh-Park et al., 2013). For instance, if a person adopts a cautious gait pattern characterized by greater stance width, then it may be possible to preserve walking speed. In contrast, if a person adopts a slower walking speed, then it may be possible to preserve stance width. Therefore, caution should be used when attempting to interpret isolated gait parameters.

The relative supply and demand of CNS resources during complex walking tasks can be expected to differ among different people (Seidler et al., 2010). This is because the relative challenge of each task will be highly dependent on the specific motor, sensory, and/or cognitive capabilities of the individual. These capabilities were not thoroughly quantified for our participants, so explaining the precise reasons for heightened CNS activity and/or diminished gait quality during complex walking tasks is outside the scope of the present study. However, we did institute a number of exclusion criteria pertaining to medical history (as described in the section “Methods”). These criteria help to ensure that our findings are not overly influenced by the presence of overt health conditions. For instance, we excluded individuals with evidence of more than mild cognitive impairment, based on the MMSE. Cognitive impairment is well known to compromise performance on tasks requiring heightened attentional demand, and may therefore strongly affect task performance. We also screened out individuals who are considered to have a heightened fall risk due to balance impairment, as indicated by the Berg Balance Test. Based on the cumulative criteria employed by our study, it is reasonable to conclude that the observed demand for CNS resources is typical of what can be expected in pre-frail older adults who are free from major medical conditions. Another factor that is known to influence performance on complex tasks is the instructions given to the participants, and the resultant prioritization of different aspects of task performance. For the present study, we gave the participants no specific information regarding how performance would be evaluated, and no instructions regarding prioritization of dual tasks. The rationale for this approach was to observe each participants natural/preferred behavior in order to maximize the relevance of our findings to actual home and community ambulation.

In summary, preparation and performance of complex walking tasks requires heightened utilization of CNS resources in older adults. The findings from this study warrant additional investigation into the role of the CNS in loss of mobility function. The non-invasive measures presented here may be valuable for identifying individuals who are at risk of adverse mobility outcomes and falls, and for gauging the effectiveness of various rehabilitation approaches.

## AUTHOR CONTRIBUTIONS

This study was designed by David J. Clark. Data collection was conducted by David J. Clark and Sarah A. Ring. Data analysis was conducted by David J. Clark and Eric C. Porges. Data interpretation was conducted by David J. Clark, Dorian K. Rose, and Eric C. Porges. The content of the manuscript was prepared by David J. Clark, Sarah A. Ring, Dorian K. Rose, and Eric C. Porges.

## Conflict of Interest Statement

The authors declare that the research was conducted in the absence of any commercial or financial relationships that could be construed as a potential conflict of interest.
